# 

**Published:** 1852-01

**Authors:** 


Ohio College of Dental Surgery.—The question has been repeatedly asked, Will Prof. Allen give the class instruction in his method of fastening teeth to plates by a cement and artificial gum? In answer, we would say, that Dr. Allen, in accepting the chair he now fills, did it with the full intention of giving the class all such instruction as properly belongs to his department, intending then, as now, and ever, so long as he holds the position of teacher in the institution, to do all in his power to advance the students, and to give them the advantage of all the knowledge he aims to make available in his own practice.

College Commencement.—The commencement exercises of the Ohio College of Dental Surgery, will take place on the evening of the 20th February, 1852, at which time the degree of Doctor of Dental Surgery will be conferred on the graduates by Rev. B. P. Aydelott, D. D., President of the board of trustees.

The graduating class will be addressed by Dr. Wright, of Pittsburgh.

The profession will be addressed by Dr. E. W. Power, of Union Town, Pa.

We hope that many of the profession at a distance will be present, and shall be glad to see also those in the city and adjacent towns on this occasion.

To the Subscribers of the Register.—We intend, with the issue of the next number of the Register, to have a tabular statement made out of the amount paid, so that each subscriber can see at once how his account stands, and we shall then continue in each successive number to notice the receipts. This will render it unnecessary to answer letters merely containing a remittance for dues. We sent the fourth volume of the Register to some fifty individuals who had not sent us their names; and in accordance with a suggestion given in the first number of that volume, we have considered all such as subscribers, except two or three who sent it back.

Our Exchanges.—We are pleased to make exchanges with good literary periodicals as well as dental and medical—perhaps there is no other way in which we so fully feel a return for our labor. Such will please address "the Dental Register, Cincinnati, Ohio."

EIGHTH ANNUAL ANNOUNCEMENT OF

### THE OHIO COLLEGE OF DENTAL SURGERY.

### SESSION OF 1852--'53.

The regular course of Lectures in this Institution commences the first Monday of November and closes on the 20th of February.

FACULTY:

**JAMES TAYLOR, M.D., D.D.S.,**
*Professor of the Principles and Practice of Dental Surgery.*

**THOMAS WOOD, M.D.,**
*Professor of Anatomy amd Physiology.*

**GEORGE MENDENHALL, M.D.,**
*Professor of Pathology and Therapeutics.*

**JOHN ALLEN, D.D.S.**
*Professor of Operative and Mechanical Dentistry.*

**G. L. VAN EMON, A.M., D.D.S.,**
*Lecturer on Dental Chemistry and Demonstrator of Operative and Mechanical Dentistry.*

The Faculty, in issuing this their eighth annual announcement, take pleasure in stating that the Institution is established on a permanent basis, and is in a flourishing condition. They feel that the difficulties which present themselves in the establishment of a new Institution of the kind, have been overcome, and that now a systematic course of instruction has been obtained which shall meet the wants and necessities of the profession.

A building, suitable in every respect for the purposes of a Dental College, is now owned by the profession and dedicated to the advancement of Dental Science.

The College buildings contain, first, a Lecture Room sufficiently large for the accomodation of one hundred and fifty students. The Dissecting Rooms are large, well lighted and ventilated. The Laboratory is large and commodious, and the Infirmary Rooms and Museum are in every respect well adapted for the purpose to which they are appropriated. Every arrangement has been made, which experience has suggested, for the good of the student, and the faculty are determined to do all in their power for the advancement of the student, not only in the practical details of the profession, but also in those branches of medical and surgical science which are so essential to the intelligent and successful dental practitioner.

A fund has been appropriated for the purchase of a Library, and such apparatus as may from time to time appear necessary.

The arrangements in the Laboratory are such as to afford every facility in Mechanical Dentistry, and a demonstrator of experience and ability takes charge of this department.

The committee for the examination of the graduating class will be selected at the close of the present session, and notice given hereafter.

| | |
|---|---|
| Tickets for the entire course, including Matriculation fee, | $100 00 |
| Diploma fee, - - - - - - - - - - - | 25 00 |

Good board can be had from $2 to $3 per week.

JAMES TAYLOR, *Dean.*

